# Src-mediated regulation of the PI3K pathway in advanced papillary and anaplastic thyroid cancer

**DOI:** 10.1038/s41389-017-0015-5

**Published:** 2018-02-28

**Authors:** Thomas C. Beadnell, Kelsey W. Nassar, Madison M. Rose, Erin G. Clark, Brian P. Danysh, Marie-Claude Hofmann, Nikita Pozdeyev, Rebecca E. Schweppe

**Affiliations:** 1 Department of Medicine, Division of Endocrinology, Metabolism, and Diabetes, University of Colorado School of Medicine, Aurora, CO 80045 USA; 2 Medical Oncology, University of Colorado School of Medicine, Aurora, CO 80045 USA; 3 Department of Endocrine Neoplasia and Hormonal Disorders, The University of Texas MD Anderson Cancer Center, Houston, TX USA; 4 University of Colorado Cancer Center, University of Colorado School of Medicine, Aurora, CO 80045 USA

## Abstract

Advanced stages of papillary and anaplastic thyroid cancer continue to be plagued by a dismal prognosis, which is a result of limited effective therapies for these cancers. Due to the high proportion of thyroid cancers harboring mutations in the MAPK pathway, the MAPK pathway has become a focal point for therapeutic intervention in thyroid cancer. Unfortunately, unlike melanoma, a similar responsiveness to MAPK pathway inhibition has yet to be observed in thyroid cancer patients. To address this issue, we have focused on targeting the non-receptor tyrosine kinase, Src, and we and others have demonstrated that targeting Src results in inhibition of growth, invasion, and migration both in vitro and in vivo, which can be enhanced through the combined inhibition of Src and the MAPK pathway. Therefore, we examined the efficacy of the combination therapy across a panel of thyroid cancer cell lines representing common oncogenic drivers (*BRAF*, *RAS*, and *PIK3CA*). Interestingly, combined inhibition of Src and the MAPK pathway overcomes intrinsic dasatinib resistance in cell lines where both the MAPK and PI3K pathways are inhibited, which we show is likely due to the regulation of the PI3K pathway by Src in these responsive cells. Interestingly, we have mapped downstream phosphorylation of rpS6 as a key biomarker of response, and cells that maintain rpS6 phosphorylation likely represent drug tolerant persisters. Altogether, the combined inhibition of Src and the MAPK pathway holds great promise for improving the overall survival of advanced thyroid cancer patients with BRAF and RAS mutations, and activation of the PI3K pathway and rpS6 phosphorylation represent important biomarkers of response for patients treated with this therapy.

## Introduction

Poorly differentiated and anaplastic thyroid cancers are characteristic of poor overall survival with an average of 3.2 and 0.5 years, respectively^1^. These cancers harbor a high prevalence of mutations in the MAPK pathway, however, in comparison to melanoma advanced thyroid cancers exhibit a reduced responsiveness to MAPK pathway inhibition^2–6^. This lack of sensitivity therefore generates the need to characterize additional pro-tumorigenic pathways in thyroid cancer, which could potentially lead to more effective therapies using additional inhibitors.

Our work and the work of others have highlighted a role for the non-receptor tyrosine kinases Src Family Kinases (Src) in mediating thyroid tumorigenesis^7–9^, and importantly we have demonstrated that prolonged inhibition of Src reprograms cells to become more reliant on the MAPK pathway^10^. In support of this data, multiple laboratories have demonstrated that the combined inhibition of Src and the MAPK pathway results in synergistic inhibition of growth and increased apoptosis of thyroid cancer cells both in vitro and in vivo^10–12^. Altogether, these data support the hypothesis that Src may mediate resistance to MAPK pathway targeted therapies, and that co-targeting Src and the MAPK pathway will result in enhanced clinical responsiveness. Having previously observed efficacy with the combined inhibition of Src and MEK1/2, in cell lines sensitive to the Src inhibitor dasatinib^10^, we hypothesized that dasatinib-intrinsically resistant cell lines would also respond to combined Src and MEK1/2 inhibition.

In the present study, we identified a correlation between increased signaling in the PI3K pathway and resistance to Src inhibition, and demonstrate that combined Src and MAPK pathway inhibition overcomes intrinsic resistance to dasatinib in BRAF-mutant and RAS-mutant cell lines, while PIK3CA-mutant cell lines tend to be more resistant. Importantly, Src appears to be a primary regulator of the PI3K pathway in BRAF-mutant and RAS-mutant thyroid cancer cell lines, which is consistent with a reduction in this pathway upon inhibition of Src or the combined inhibition of Src and MEK1/2. Consistent with this data, previous work has shown that Src regulates the PI3K pathway through different effectors including PTEN, p85, and AKT^13–17^.

Multiple studies have now demonstrated enhanced efficacy of targeting the MAPK pathway in combination with either Src or PI3K pathway inhibitors in thyroid cancer^10–12,18–21^, and importantly, our data suggests that Src can also regulate the PI3K pathway in thyroid cancer. Therefore, the identification of downstream biomarkers of inhibitor response will provide important indications for the effectiveness of these combination therapies. Herein, we demonstrate that cells sensitive to the combined inhibition of both Src and the MAPK pathway exhibit an enhanced reduction in rpS6 phosphorylation, whereas cell lines resistant to the combination therapy maintain rpS6 phosphorylation to a greater extent. Surprisingly, even cell lines that exhibit increased sensitivity to the combination therapy still maintain a small subset of cells that maintain rpS6 phosphorylation suggesting that these cells may give rise to drug tolerant persisters. Taken together, these studies provide additional rationale for the further identification of the signaling mechanisms that mediate thyroid cancer growth and survival, which will ultimately lead to more effective elimination of drug tolerant persister cells, and an enhanced overall survival for patients with advanced stages of thyroid cancer.

## Results

### Activation of the PI3K pathway is associated with dasatinib intrinsic resistance

**Fig. 1:**
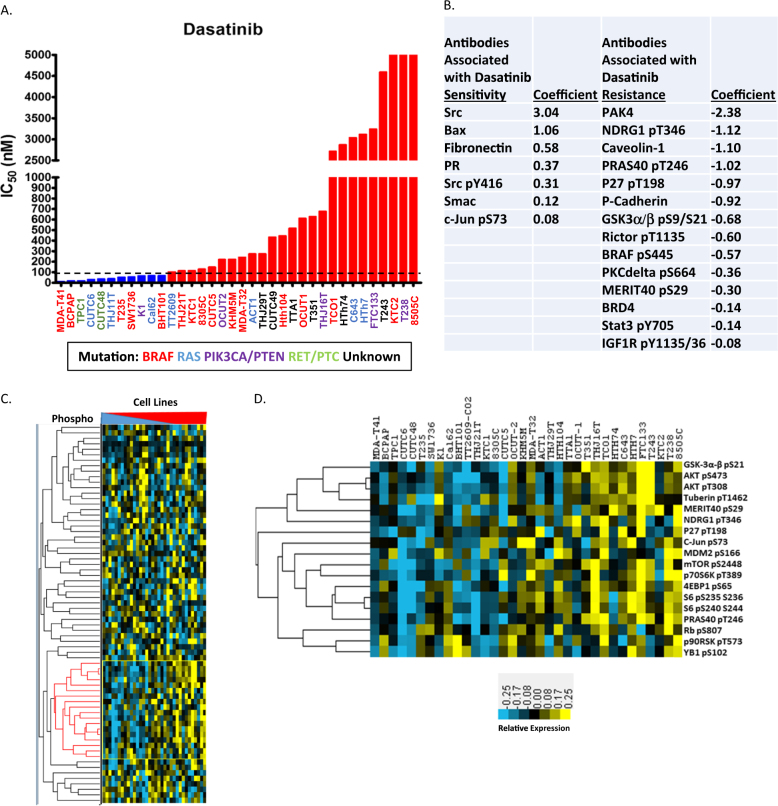
Analysis of dasatinib intrinsic sensitivity and resistance. **a** Absolute quantification of the IC_50_ value for dasatinib, in 36 thyroid cancer cell lines. Growth curves were measured across the cell lines using the CellTiter-Glo Assay (Promega), and the IC50 values were calculated. An IC_50_ cutoff of 90 nM (dashed line) was used to determine cell lines sensitive and resistant to dasatinib. **b** Protein expression parameters associated with dasatinib sensitvity and resistance in elastic net regression analysis of RPPA data generated from 34 thyroid cancer cell lines. Higher absolute coefficient value illustrates stronger association with dasatinib sensitivity or resistance. **c** Hierarchical clustering of phosphoprotein expression data obtained by RPPA analysis of 34 thyroid cancer cell lines. Cell lines were arranged in increasing dasatinib IC_50_ order from most sensitive to most resistant. **d** phosphoprotein cluster containing many components of PI3K pathway, whose expression positively correlates with dasatinib resistance

To identify thyroid cancer cell lines sensitive or resistant to dasatinib, we analyzed the sensitivity of 36 thyroid cancer cell lines, to the Src inhibitor dasatinib, using an IC_50_ cutoff of 90 nM, which is based on the peak plasma/serum concentrations of dasatinib in patients treated for chronic myelogenous leukemia, as well as the selectivity of dasatinib (Fig. 1a)^22^. Interestingly, there appears to be an additional separation in the resistant group (90 nM), which may demarcate intermediate and higher levels of resistance. We next performed Reverse Phase Protein Analysis (RPPA) to determine signaling differences between the sensitive and resistant groups, and used elastic net regression analysis to identify proteins whose expression is associated with the sensitivity (positive coefficients) and resistance (negative coefficients) to dasatinib (Fig. 1b). The cross-validation of the elastic net model indicated that protein expression data obtained with RPPA indeed explains the sensitivity to dasatinib (elastic net model was superior to the null model) (Figure S1a). In support of this, expression of Src was the major protein associated with sensitivity to dasatinib (Fig. 1b). We next analyzed proteins enriched in cell lines resistant to dasatinib, and observed numerous proteins associated with the PI3K pathway and downstream effectors of AKT. To further delineate a correlation between PI3K pathway activation and increasing resistance to dasatinib, we performed clustering analysis of the phosphorylated antibodies quantified using RPPA. We identified a cluster of phosphoproteins, in which expression is enriched with increased resistance to dasatinib (Fig. 1c; denoted with red lines). Analysis of this cluster contained many proteins predictive of dasatinib resistance in the elastic net regression model, as well as additional components of the PI3K pathway (AKT pS473, AKT T308, mTOR pS2448, p70S6K T389, 4EBP1 pS65, S6 S235/236, and S6 S240/244) and downstream targets of AKT (Tuberin pT1462 and YB1 pS102; Fig. 1d).

### A reduction in PI3K pathway signaling correlates with sensitivity to the combined inhibition of Src and the MAPK pathway

**Fig. 2:**
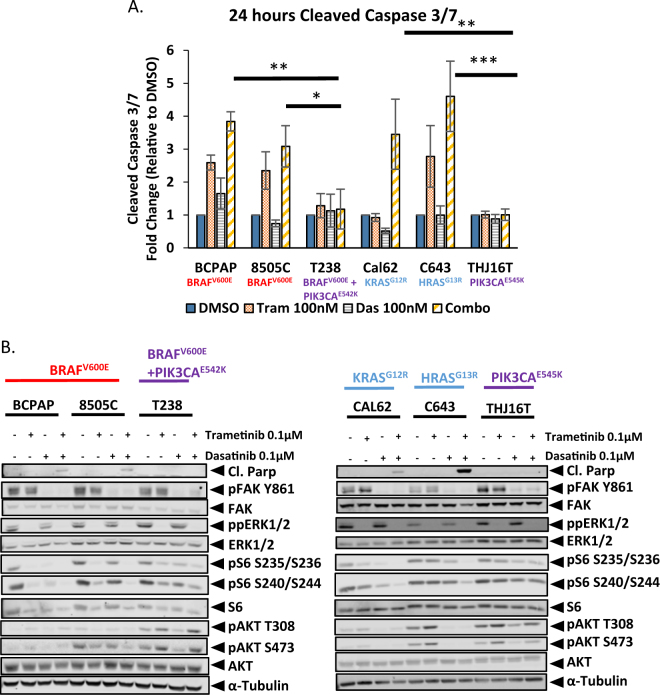
Analysis of Src and MEK1/2 inhibitor sensitivity in BRAF-mutant, RAS-mutant, and PIK3CA-mutant cell lines. **a** Cleaved caspase 3/7 activity was measured after a 24 h incubation with either DMSO, 100 nM trametinib, 100 nM dasatinib, or dasatinib+trametinib in the BCPAP, 8505C, T238, Cal62, C643, or THJ16T cell line. Data are represented as means+SEM (*n* = 3; Student’s *t*-test; *P < 0.05, **P < 0.005, ***P < 0.0005). **b** The BCPAP, 8505C, T238, Cal62, C643, or THJ16T cells were treated for 24 h with the indicated inhibitors, and then whole-cell lysates were analyzed by Western blot analysis using the indicated antibodies

On the basis of our previous work demonstrating enhanced synergy between Src and MEK1/2 inhibitors, in dasatinib sensitive cell lines^10^, we examined the efficacy of combined inhibition of Src and the MAPK pathway in cell lines intrinsically resistant to dasatinib. To test this, we chose thyroid cancer cell lines containing mutations in the commonly mutated oncogenes; BRAF, RAS, and PIK3CA (Table 1), along with two cell lines sensitive to dasatinib; the BRAF mutant, BCPAP, and the RAS-mutant, Cal62^10^. To determine sensitivity, we analyzed the induction of apoptosis, upon treatment with either single agent (100 nM trametinib or 100 nM dasatinib) or the combination (Fig. 2a). Surprisingly, only the BRAF-mutant (BCPAP and 8505C) and RAS-mutant (Cal62 and C643) cell lines, tested, regardless of their intrinsic sensitivity or resistance to dasatinib, exhibited significant 2.5 to 5-fold increases in apoptosis upon treatment with the combination therapy, in comparison to the DMSO controls (*p*-value <0.01) (Fig. 2a). Of further interest, no increase in apoptosis was observed in the PIK3CA-mutant cell lines (T238 and THJ16T; Fig. 2a). We therefore tested two additional PIK3CA-mutant cell lines, to determine if the presence of a PIK3CA-mutation promotes resistance to the combination therapy, and interestingly found the K1 cell line also exhibited resistance to the combination therapy, whereas the OCUT-2 cell line was sensitive (Figure S2A). Thus, other factors may mediate sensitivity to combined Src and MAPK inhibition in the OCUT-2 cells. Given the differential responses observed amongst the different oncogenic drivers, we next analyzed pathway signaling responses in relation to the inhibitory effects of either the single agents or the combination, to define mechanisms of response. Consistent with the apoptosis assay, we only observed an increase in Poly ADP-ribose polymerase (PARP) cleavage in the BRAF- and RAS-mutant cell lines treated with the combination therapy (Fig. 2b). In addition, we analyzed the downstream effectors of Src and MEK1/2, and observed a similar reduction in FAK Y861 and ERK1/2 phosphorylation, respectively, in all of the cell lines (Fig. 2b and Table S1). We then analyzed the phosphorylation status of the downstream target of both the MAPK and PI3K pathways, rpS6 (Fig. 2b). Interestingly, treatment with the combination therapy in the BRAF-mutant and RAS-mutant cell lines resulted in a significant 92–98 percent reductions in rpS6 S235/S236 phosphorylation (*p*-value <0.05), and 78–94 percent reductions in rpS6 S240/S244 phosphorylation, in comparison to combination treated PIK3CA-mutant cell lines, which resulted in only 59–65 percent reductions in rpS6 S235/S236 phosphorylation and 26–40 percent reductions in rpS6 S240/S244 phosphorylation (Figure S2b and Table S1). We next analyzed AKT phosphorylation, and consistent with the RPPA analysis, we observed elevated levels of AKT phosphorylation in all of the dasatinib-intrinsically resistant cell lines (Figs. 1b and 2b). We therefore analyzed the effects of Src and/or MEK1/2 inhibition on AKT phosphorylation (T308 and S473), and only observed reductions in the BRAF- and RAS-mutant dasatinib-intrinsically resistant cell lines, with 63–80 percent reductions in the 8505C and 94–95 percent reductions in the C643, cell line, in comparison to the DMSO control, respectively (Fig. 2b, S2b, and Table S1). Importantly, treatment with the combination therapy significantly reduced AKT S473 phosphorylation in the BRAF- and RAS-mutant dasatinib-intrinsically resistant cell lines, in comparison to the resistant PIK3CA-mutant cell lines and the dasatinib-intrinsically sensitive cell lines (*p*-value <0.05; Figure S2b). Taken together, these data support a hypothesis in which the combined inhibition of Src and the MAPK promotes sensitivity through inhibition of both the MAPK and PI3K pathways.

**Table 1:** Cell line characteristics

	Mutation	Type	Dasatinib	Dasatinib IC_50_ (nM)
BCPAP	BRAF (V600E)	Papillary	Sensitive	19
8505C	BRAF (V600E)	Anaplastic	Resistant	>3000
Cal62	KRAS (G12R)	Anaplastic	Sensitive	66
C643	HRAS (G13R)	Anaplastic	Resistant	>3000
T238	BRAF (V600E)/PIK3CA E542K	Anaplastic	Resistant	>3000
THJ16T	PIK3CA E545K	Anaplastic	Intermediate	677

Cell lines are categorized based on mutation status, tumor subtype, and sensitivity or resistance to dasatinib. Sensitivity is based on an IC_50_ value <90 nM, as determined by CellTiter-Glo assay (Promega; Fig. 1a)

Finally, we also wanted to retrospectively analyze tumors generated from the RAS-mutant cell line, Cal62, in a mouse model of thyroid tumorigenesis, which when treated with the dasatinib/trametinib combination therapy resulted in enhanced overall survival^10^. From this study, tumors were treated with either the vehicle, trametinib, dasatinib, or the combination. Interestingly, both pS6 S235/236 and pSRC Y416 levels were reduced with the combination therapy in comparison to either the vehicle or the single-agent inhibitors (Figure S3). Phosphorylation of ERK1/2 exhibited similar inhibition with both trametinib and the combination therapy (Figure S3). Thus, this data supports rpS6 as biomarker of response to the combined inhibition of Src and the MAPK pathway in an in vivo model of thyroid tumorigenesis.

### Expression of a drug-resistant c-Src attenuates the growth inhibitory effects of the combined inhibition of Src and the MAPK pathway

**Fig. 3:**
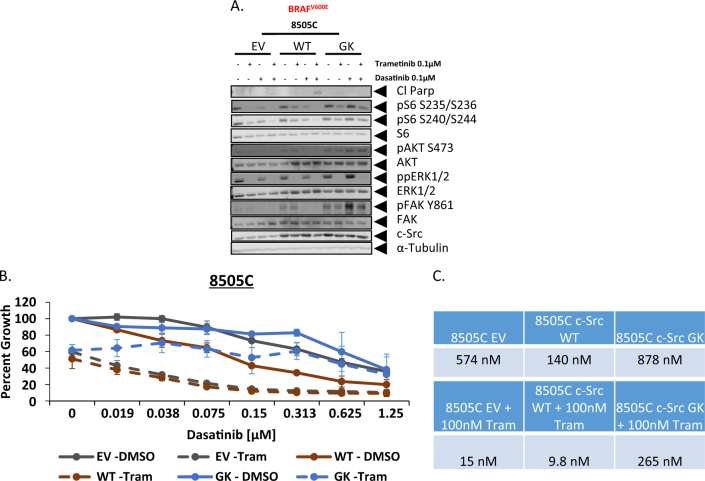
Inhibition of Src is required for the combined inhibitory effects of dasatinib and trametinib. **a** Western blot analysis was performed on whole cell lysates from the 8505C cell line expressing either an empty vector (EV), wild-type Src (WT) or drug-resistant gatekeeper mutant Src (GK) treated with either DMSO, 100 nM trametinib, 100 nM dasatinib, or the combination for 24 h. Lysates were analyzed using the indicated antibodies. **b** The 8505C cell line expressing either an empty vector (EV), wild-type Src (WT) or drug-resistant GK mutant Src were treated with increasing doses of dasatinib ranging from (0.019 μM to 1.25 μM) for 72 h, in the presence of either DMSO or 100 nM trametinib. Cell growth was measured using the Sulforhodamine B assay. **c** Dasatinib IC_50_ values were calculated using graphpad prism software in the presence or absence of 100 nM trametinib

Due to the ability of dasatinib to target a number of kinases, in addition to Src^23,24^, we wanted to next validate the role of Src in mediating tumorigenesis and regulating the PI3K pathway, in the dasatinib-intrinsically resistant cell lines. To accomplish this, we transduced the c-SRC drug-resistant gatekeeper mutant (c-Src T338I) (GK), as well as c-SRC wild-type (WT), and empty vector (EV) constructs into the BRAF-mutant dasatinib-intrinsically resistant cell line, 8505C. Comparison of the three constructs demonstrates that the c-Src GK mutant maintains FAK Y861 phosphorylation in the presence of dasatinib or the combination, but does not prevent the reduction in ERK1/2 phosphorylation observed with either single-agent trametinib or the combination (Fig. 3a). Importantly, the c-Src GK mutant abrogates the reduction in AKT and rpS6 phosphorylation observed with either single agent or the combination, indicating that the ability of dasatinib to reduce signaling through the PI3K pathway is mediated through its inhibitory effects on Src (Fig. 3a). We next analyzed the ability of the c-Src GK mutant to promote resistance. Consistent with Src expression being associated with dasatinib sensitivity in regression analysis, cells expressing exogenous WT Src exhibited enhanced dasatinib sensitivity (IC_50_ = 140 nM), in comparison to the c-Src GK mutant (IC_50_ = 878 nM), and empty vector (IC_50_ = 575 nM) constructs (Fig. 3c). Consistent with there being no difference in the ability of trametinib treatment to reduce ERK1/2 phosphorylation (Fig. 3a), we also observed no difference in growth reduction, upon treatment with single-agent MEK1/2 inhibition (Fig. 3c). Importantly, when cells were treated with the combination of dasatinib and tramentinib, the c-Src GK mutant cells (IC_50_ = 265 nM) exhibited increased resistance in comparison to the WT (IC_50_ = 9.8 nM) and empty vector (IC_50_ = 15 nM) expressing cells (Fig. 3c). Therefore, this data supports a role for Src-mediated PI3K pathway activation, in thyroid cancer cell lines.

### rpS6 phosphorylation does not play a functional role in mediating the growth inhibitory effects of the combined inhibition of Src and the MAPK pathway

**Fig. 4:**
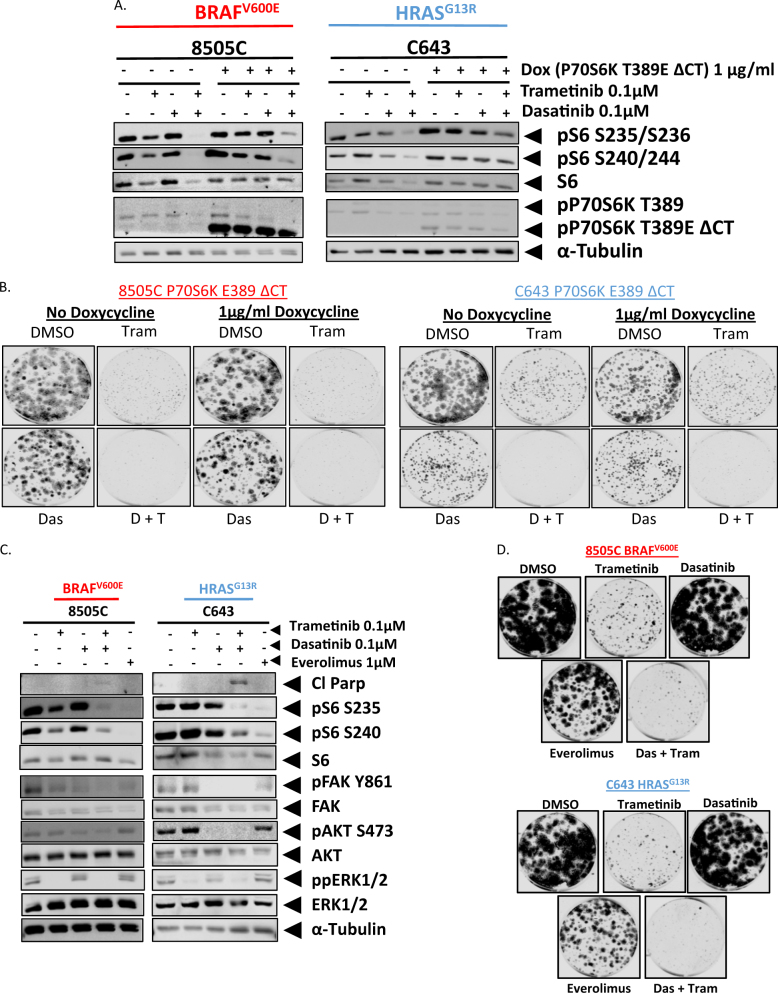
rpS6 phosphorylation is neither necessary nor sufficient for the growth inhibitory effects of the combined inhibition of Src and the MAPK pathway. **a** Western blot analysis was performed on whole cell lysates from the 8505C or C643 cell line expressing a doxycycline inducible P70S6K T389 ΔCT construct. Cells were treated with or without doxycycline for 24 h prior to treatment. After 24 h the cells were treated with either DMSO, 100 nM trametinib, 100 nM dasatinib, or the combination of dasatinib and trametinib. Lysates were analyzed for the indicated antibodies. Quantification was performed using the Odyssey Image Studio V.4.0 Software. Data as means±SEM (*n* = 3). **b** Clonogenic growth was detected by crystal violet staining in the 8505C or C643 cell lines expressing the doxycycline inducible P70S6K T389 ΔCT construct. Cell lines were treated with or without doxycycline upon plating. 24 h later the cell lines were treated with either DMSO, 100 nM trametinib, 100 nM dasatinib, or the combination for 6 days. Following 6 days of treatment, the cells were released for an additional 7 days. **c** Western blot analysis was performed on whole cell lysates from the parental 8505C or parental C643 cell lines treated with either DMSO, 100 nM trametinib, 100 nM dasatinib, or 1 μM Everolimus for 24 h for the indicated antibodies. Quantification was performed using the Odyssey Image Studio V.4.0 Software. Data as means±SEM (*n* = 3). **d** Clonogenic growth was detected by crystal violet staining in the 8505C and C643 cell lines. Cell lines were treated for 24 h with either DMSO, 100 nM trametinib, 100 nM dasatinib, dasatinib+trametinib, or 1 µM everolimus. Colony area signal intensity was measured using Odyssey CLx imager (Li-Cor), and presented as percent fold change relative to the DMSO treated wells. Data as means±SEM (*n* = 3)

Taken together, our data indicates a role for phospho-rpS6 in mediating sensitivity and resistance to the combined inhibition of Src and the MAPK pathway. To test this hypothesis, we expressed a doxycycline inducible, constitutively active, P70S6K-E389-ΔCT (P70S6K-CA), construct into the BRAF-mutant and RAS-mutant dasatinib-intrinsically resistant cell lines, 8505 C and C643, respectively^25,26^. The addition of doxycycline induced the expression of P70S6K-CA (Fig. 4a). Expression of P70S6K-CA was able to partially rescue the inhibition of rpS6 phosphorylation in the presence of the combination therapy, as we observed a 2-fold to 5-fold attenuation in the reduction in rpS6 phosphorylation when treating with the combination therapy, in comparison to the controls. (Fig. 4a and S4a). Importantly, doxycycline treatment alone does not impact the inhibition of S6 phosphoryaltion (Figure S4c). Therefore, we next analyzed the ability of P70S6K-CA to prevent a reduction in clonogenic growth in response to either single agent (trametinib or dasatinib) or the combination. Despite a rescue in rpS6 phosphorylation, expression of P70S6K-CA was unable to rescue the growth inhibitory effects of the combination therapy or either single agent alone (Fig. 4b and S4b).

We next asked whether a reduction in rpS6 phosphorylation is sufficient to induce the increase in apoptosis observed with the combination therapy. Treatment of the dasatinib intrinsically resistant cell lines (8505C and C643) with the MTORC1 inhibitor everolimus, resulted in a 3-10 fold enhanced reduction in rpS6 phosphorylation at the S235/S236 site in comparison to the combined inhibition of dasatinib and trametinb, however only the combination therapy was able to induce an increase in apoptosis (Fig. 4c and S4d). As expected, no reduction in FAK, ERK, or AKT phosphorylation was observed with everolimus, whereas we did observe inhibition of these targets with Src and/or MEK1/2 inhibition (Fig. 4c). In addition, the combination therapy was able to more effectively inhibit clonogenic growth in comparison to everolimus (Fig. 4d and S4e). Taken together, these results indicate that the effectors of the PI3K pathway that are necessary for growth and survival are upstream of P70S6K and downstream of Src

### Heterogeneous phospho-rpS6 Responses to single-agent and combination therapy

**Fig. 5:**
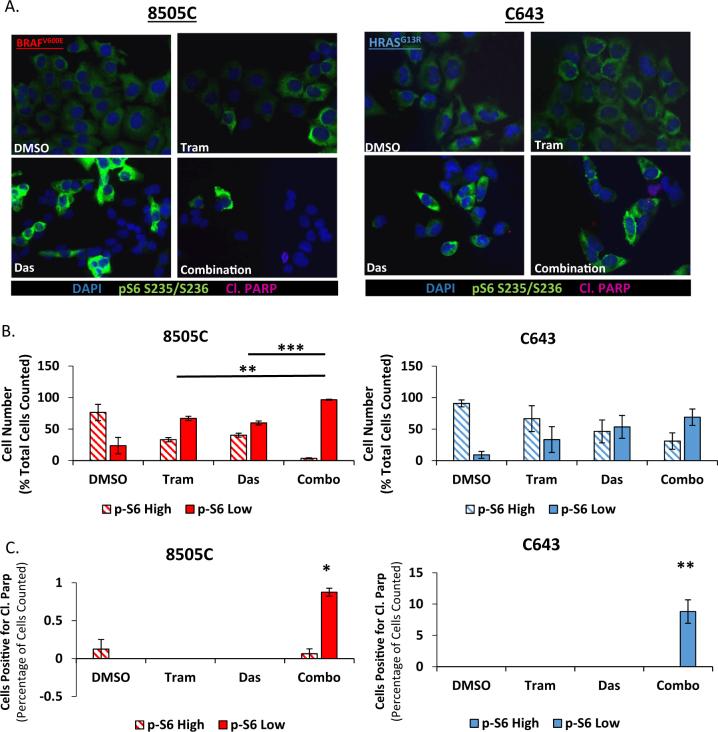
Single cell analysis identifies heterogeneous phospho-rpS6 responses to single-agent and combination therapy. **a** the BRAF-mutant, 8505C, and RAS-mutant, C643, cell lines were analyzed by immunofluorescence for pS6 S235/S236 (Green), PARP Cleavage (Red), and Dapi (Blue) after 24 h of treatment with either DMSO, 100 nM trametinib, 100 nM dasatinib, or the combination. **b** Individual cells were analyzed for each treatment group for their intensity levels of S6 phosphorylation. P-S6 low represents signal intensity below half of the average intensity for the DMSO treated cells, and p-S6 high represents any cells expressing greater than half of the average intensity for the DMSO treated cells. Data as means +/− SEM (*n* = 3; Student’s *t*-test; **P < 0.005; ***P < 0.0005). **c** Average percent cleaved PARP positive cells for both the p-S6-high and p-S6-low groups were normalized to total treatment group cell count and averaged across three individual experiments. Significance is calculated in relation to the DMSO treated p-S6 high values. Data as means ±SEM (*n* = 3; Student’s *t*-test; *P < 0.05; **P < 0.005)

Despite an enhanced sensitivity to the combination therapy in BRAF-mutant and RAS-mutant cell lines, the combination therapy is unable to completely eliminate colony formation (Fig. 4b, d). Therefore, to better define the role of rpS6 phosphorylation status, as a biomarker of response to the combination therapy, we analyzed rpS6 phosphorylation at the single cell level in the two dasatinib-intrinsically resistant cell lines that respond to the combination therapy, 8505C and C643. Surprisingly, treatment with either single-agent or the combination therapy did not result in a universal reduction in rpS6 phosphorylation (Fig. 5a, b). Rather, a subset of cells remain after treatment that do not exhibit a reduction in rpS6 phosphorylation (phospho-rpS6 persisters). Thus we next quantified the percentage of cells exhibiting phospho-rpS6 (p-S6) low or high expression. In the BRAF-mutant, 8505C, cell line, MEK1/2 or Src inhibition resulted in a 43 and 36% increase in p-S6 low expressing cells in comparison to the DMSO treated cells, respectively, and the combination therapy resulted in a significant 73% increase in cells with p-S6 low expression, in comparison to both MEK1/2 and Src inhibition alone (*P* = 0.0011 and *P* = 0.0003, respectively) (Fig. 5b). In the RAS-mutant, C643, cell line, MEK1/2 and Src inhibition resulted in a 24% and 44% increase in p-S6-low expressing cells, in comparison to the DMSO control. Importantly, the combination therapy resulted in a 60% increase in p-S6-low expressing cells (Fig. 5b). We next analyzed cells for evidence of cell death, by quantifying cells positive for PARP cleavage. Similar to western blot analysis, PARP cleavage was primarily observed in cells treated with combined Src and MAPK pathway inhibitors (Fig. 5c and Table S3). Importantly, in cells treated with the combination therapy, there was a significant increase in cleaved PARP positive cells in pS6 low expressing cells, in comparison to the DMSO treated p-S6 high expressing cells (8505C; *P* = 0.0053 and C643; *P* = 0.0093; Fig. 5c). We also observed PARP cleavage in 1 DMSO and 1 combination treated 8505C cell that exhibited p-S6 high expression, however as these results were inconsistent, we did not observe a significant correlation. Taken together, these data suggest that rpS6 phosphorylation may be a biomarker of response for cancer cells that are responsive to the combined inhibition of Src and the MAPK pathway.

### Analysis of AKT inhibition in relation to Src and MEK1/2 inhibition

**Fig. 6:**
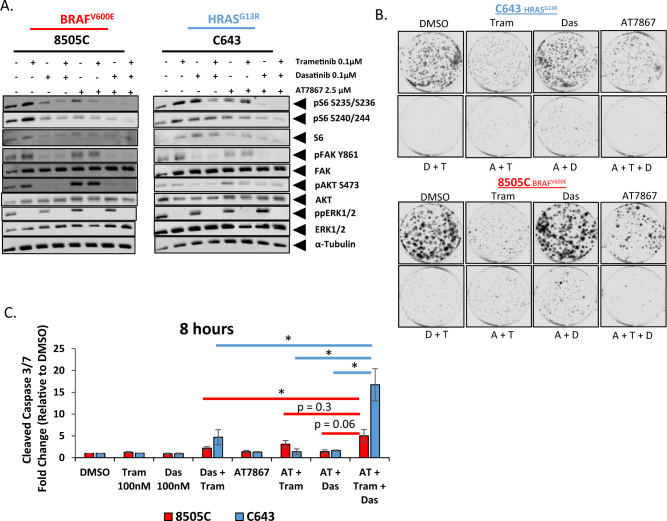
Combined Src, MEK1/2 and AKT/P70S6K inhibition more effectively reduces rpS6 phosphorylation and potentiates inhibitor effects. **a** Western blot analysis was performed on whole cell lysates from the 8505C and C643 cell lines treated with either DMSO, 100 nM trametinib, 100 nM dasatinib, dasatinib+trametinib, 2.5 μM AT7867, trametinib+AT7867, dasatinib +AT7867, or trametinib+dasatinib+AT7867 for 4 h and analyzed for the indicated antibodies. **b** Clonogenic growth was detected by crystal violet staining in the 8505C or C643 cell lines. The cells were treated with either DMSO, 100 nM trametinib, 100 nM dasatinib, 2.5 μM AT7867, trametinib+AT7867, dasatinib+AT7867, or trametinib+dasatinib+AT7867 for 3 days. Following 3 days of treatment, the cells were released for an additional 6 days. Colony area signal intensity was measured using Odyssey CLx imager (Li-Cor), and presented as percent fold change relative to the DMSO-treated wells. Data as means±SEM (*n* = 2). **c** Cleaved caspase 3/7 activity was measured after an 8 h incubation with either DMSO, 100 nM trametinib, 100 nM dasatinib, dasatinib+trametinib, 2.5 μM AT7867, trametinib+AT7867, dasatinib+AT7867, or trametinib+dasatinib+AT7867 in the 8505C or C643 cell line. Data as means±SEM (*n* = 3; Student’s *t*-test; *P < 0.05)

Having observed that a subset of the 8505C and C643 cells maintain rpS6 phosphorylation upon treatment with the combination therapy (Fig. 5b), we hypothesized that more effective inhibition of rpS6 phosphorylation may potentiate the cytotoxic effects in these cell lines. On the basis of our observations in Fig. 2, in which PIK3CA-mutant cell lines were resistant to the combination therapy, we next tested the effects of Src and MEK1/2 inhibition in combination with an AKT/P70S6K inhibitor, AT7867. Due to the high potency of this triple combination, a shorter (4 h) time point was used for this experiment. Interestingly, in contrast to longer treatments (24 h in Figs. 2 and 4), single-agent treatment with trametinib for 4 h results in higher levels of pS6, pFAK, and pAkt in the 8505C cells, and pS6-S240/244 in the C643 cells. Nonetheless, combined inhibition of both the MAPK and the PI3K pathways, with trametinib and dasatinib, with or without AT7867, respectively, correlates with reduced rpS6 phosphorylation and enhanced inhibition of growth and increased apoptosis (Fig. 6a–c). Important for future considerations, is the observation that the combined inhibition of Src and AKT/P70S6K results in the largest decrease in rpS6 phosphorylation in comparison to the other dual inhibitory combinations (Fig. 6a and S5a), however this decrease in rpS6 phosphorylation does not correlate with a similar increase in apoptosis or a decrease in cell growth (Fig. 6b, c and S5b). Taken together, rpS6 phosphorylation status appears to be an important indicator for the effectiveness of combined MAPK and Src/PI3K pathway inhibition.

## Discussion

Src-driven oncogenesis has been well documented in a number of cancers, however unlike typical oncogenic drivers, Src is rarely mutated^27,28^. We previously demonstrated that chronic Src inhibition promotes an increased reliance of thyroid cancers on the MAPK pathway, and that the upfront combined inhibition of Src and the MAPK pathway can prevent/delay resistance from occurring^10^. Our study, along with others highlighting synergy between the combined inhibition of Src and the MAPK pathway, support a role for Src in mediating resistance to inhibitors of the MAPK pathway, in thyroid cancer^10–12^. Therefore, we set out to determine which patients would benefit from the combined inhibition of Src and the MAPK pathway.

Herein, we provide evidence to support a role for Src in mediating activation of the PI3K pathway, and the ability to inhibit PI3K signaling correlates with sensitivity to combined Src and MAPK pathway inhibition. In support of this, Src has been demonstrated to drive resistance to prolonged B-Raf inhibition in melanoma^29^. A role for Src in mediating PI3K pathway activation also importantly aligns the combined inhibition of Src and the MAPK pathway with additional studies in thyroid cancer analyzing the combined inhibition of the MAPK and PI3K pathways^21,30^. In addition, a recent salvage therapy case for a patient with anaplastic thyroid cancer treated with the B-Raf inhibitor, dabrafenib, and the MEK1/2 inhibitor, trametinib, utilized rpS6 phosphorylation status as an indicator for a lack of responsiveness to this therapy, which prompted the addition of the mTOR inhibitor, everolimus, which promoted a dramatic regression in tumor volume^31^. Indeed, multiple studies in different tumor types have also highlighted the efficacy of targeting both the MAPK and PI3K pathway^32–35^. Taken together, these data support rpS6 as an important indicator of the effective reduction in MAPK and PI3K pathway activation. However, as we only analyzed growth and apoptosis in this study, the role of Src in mediating invasion and migration warrants further exploration, as there may be additional benefits from targeting Src, and inhibiting the metastatic potential of thyroid cancer cells^36^.

A previous report form Axelrod et al.^37^, demonstrated that overexpression of a constitutively active P70S6K construct is able to overcome sensitivity to the combined inhibition of the HER-family kinases and AKT, through the maintenance of rpS6 phosphorylation. Surprisingly, we did not observe similar results with dasatinib and trametinib, which may be due to a difference in the mutational landscape of the cell lines tested, as the aforementioned study did not analyze cell lines with BRAF or RAS mutations. In addition, rpS6 is not the only node of convergence between the MAPK and PI3K pathways, as additional effectors include the forkhead box O (FOXO) family members, c-myc, BCL2-associated agonist of cell death (BAD), GSK3, as well as others^38^. Therefore, our data demonstrates that there may be alternative effectors mediating the oncogenic signaling outputs derived from the MAPK and PI3K pathways, and that these alternative effectors should be analyzed in more detail to determine the key downstream nodes mediating growth and survival. Interestingly in support of this, the majority of PIK3CA-mutant thyroid cancer cell lines tested did not exhibit an increase in apoptosis in response to combined inhibition of Src and the MAPK pathway. Furthermore, this is not completely surprising as our findings in Fig. 1b uncovered an association between elevated levels of the pro-apoptotic proteins BAX and SMAC being associated with Src inhibitor sensitivity, suggesting that a link between Src and PI3K pathway signaling can be uncoupled through the acquisition of PI3K mutations.

Despite rpS6 lacking a functional role in our cell lines in response to the combination therapy, we nonetheless found that rpS6 represents an important biomarker of response to combined inhibition of Src and the MAPK pathway. Importantly, phospho-rpS6 persister cells have the potential to give rise to a drug tolerant population of cells within tumors treated with this combination therapy. Herein, we found that additional activity in the PI3K pathway may be mediating the survival of these phospho-rpS6 persisters. Along these lines, the addition of a PI3K pathway inhibitor to the combination resulted in enhanced inhibition of growth and apoptosis. Interestingly, differential signaling results were observed between 24 and 48 h treatments for single-agent inhibitors, which may be important for future consideration in regards to mechanisms of reprograming that underlie thyroid cancer sensitivity and cell death, in response to the combination therapies. PI3K isoforms have previously been demonstrated to be alternatively regulated^39,40^, which provides rationale that Src may selectively regulate a specific PI3K isoform that can be compensated for by an alternative isoform^41,42^. In addition, differences in the mutational landscape of thyroid cancer cell lines, especially in regards to passenger mutations in the PI3K pathway, may also play a role in regulating differential sensitivity to therapies. Future studies will therefore aim to define the differential roles of PI3K isoforms and secondary PI3K pathway mutations in mediating thyroid cancer cell growth and survival, and determine the role of Src in conjunction with these isoforms and alternative pathway mutations.

Taken together, our data provides a critical piece of information in regards to the role of Src in thyroid cancer, and the patient populations most likely to respond to the combined inhibition of Src and the MAPK pathway. In addition, the identification of rpS6 phosphorylation as a biomarker of response to the combined inhibition of Src and the MAPK pathway provides an important tool for the identification of drug tolerant persisters, and the future determination of pathways that mediate resistance to this combination strategy. Finally, we predict that the discoveries presented in this study will pave the way for improved responses, and enhanced overall survival for patients with advanced papillary and anaplastic thyroid cancer.

## Materials and methods

### Reagents

For the drug screening assays, AT7867 and Everolimus were purchased from SelleckChem, trametinib (GSK-1120212) and dasatinib (BMS-354825) were purchased from LC laboratories. The drugs were dissolved in dimethyl sulfoxide.

### Cell Culture

Human thyroid cancer cell lines C643, BCPAP, T238, THJ16T, 8505C, and Cal62 were grown in RPMI (Invitrogen) supplemented with 5% FBS (HyClone Laboratories). Cell lines used for drug screening and RPPA analysis are included in Supplemental Table 2^43–45^, and were supplemented with 5% FBS (HyClone Laboratories). All lines were maintained at 37 °C in 5% CO_2_. Cell lines were validated using STR profiling using the Applied Biosystems Identifier kit (#4322288) in the Barbara Davis Center BioResources Core Facility, at the University of Colorado, as previously described^43^. The C643 cells were generously provided by Dr. K. Ain, with permission from Dr. N.E. Heldin. The BCPAP, 8505C, and Cal62 cells were generously provided by Dr. M. Santoro. The THJ16T cells were generously provided by J.A. Copland. All cell lines were routinely monitored for *Mycoplasma* contamination using the Lonza Mycoalert system (Lonza). *BRAF, RAS*, and *PIK3CA* mutational status was confirmed by Sequenom and/or SANGER sequencing analysis (data not shown).

### Cellular Growth Assays

#### CellTiter-Glo 2.0 Assay (Promega)

Cells (100–500/well) were plated in opaque-walled 396-well plates, in 25 μl of RPMI media with 5% FBS. Cells were then treated with 8 concentrations of dasatinib (0.64–40,000 nM) for 72 h and cell proliferation was measured using CellTiterGlo 2.0 assay following manufacturer’s protocol. Luminescence output was analyzed in *R* and dose-response curve parameters (including IC_50_) were estimated using 4-parameter log-logistic regression.

#### Sulforhodamine B Assay

Cells (1000–1500/well) were plated in triplicate in 96-well plates. Cells were treated with increasing concentrations of the indicated drugs and cell growth was measured by SRB assay after 3 days of drug treatment, as previously described^7,46^.

#### Clonogenic Assay

Cells (1000) were plated in 6-well plates and treated with indicated inhibitors every 3 days for 6 days. On day 6, the cells were washed and released from treatment for an additional 7 days. For experiments involving the AT7867 inhibitor, cells were treated for 3 days and were released for 6 days. Cells were stained with crystal violet, and imaged and analyzed using the Odyssey CLx imager (Li-Cor), as previously described^10^.

### Cellular Apoptotic Assay

Cells were plated in duplicate, in 96-well plates, and allowed to adhere overnight. Media was replaced with RPMI containing 0.1% FBS, and 6 or 22 h later the cells treated with indicated inhibitors for either 24 or 8 h. Cleaved caspase 3/7 luminescence was measured using the caspase-glo 3/7 assay (Promega) using the Synergy H1 hybrid plate reader (Biotek).

### Immunoblotting

Cells were collected in CHAPs lysis buffer (containing 10 mmol/L CHAPs, 50 mmol/L Tris (pH 8.0), 150 mmol/L NaCl, and 2 mmol/L EDTA with 1× protease/phosphatase inhibitor cocktail (Thermo). Protein (20 μg) was separated using an 8% PAGE-SDS gel, and transferred to Immobilon-P membranes (Millipore). Membranes were incubated overnight at 4 °C with the indicated antibodies. Antibodies were purchased from Cell Signaling: pAKT-S473 (9271), pAKT-T308 (4056), AKT (2920), ppERK1/2 (4370), ERK1/2 (9107), pP70S6K (9234), pS6-S235 (4858), pS6-S240 (5364), S6 (2317), and c-Src (2123), Life Technologies: pFAK—Y861 (44–626G), BD Biosciences: Cl. Parp (552596) and FAK 610087), and CalbioChem: α-Tubulin (CP06). Blots were incubated with indicated antibodies and imaged and quantified using the Odyssey Clx imager (Li-Cor).

### Viral transfections and generation of stable cell lines

#### c-Src WT and GK

8505 C and C643 cell lines were transduced with pBABE-EV-hygro, pBABE-WT-c-SRC (Addgene plasmid 26983), or pBABE-GK-c-Src T338I (Addgene plasmid 26980) retrovirus and selected with hygromycin 0.5 mg/ml or 0.2 mg/ml, respectively, as previously described^7^.

#### P70S6K-E389

The pSLIK S6K (E389-deltaCT) neo was a gift from Kevin Janes (Addgene plasmid #58516). The construct was packaged for lentiviral delivery via HEK293FT cells using Effectene transfection reagent (Qiagen), and cells were transduced and selected with G418 0.5 mg/ml.

### Immunofluorescence

Cells were seeded at a density of 20,000 cells/well, and treated with indicated inhibitors for 24 h. Cells were then fixed with 2% PFA, permeabilized in methanol, Blocked with Odyssey blocking buffer (PBS) (Leicor), and then incubated with the primary antibodies diluted in Odyssey blocking buffer (PBS) overnight at 4 °C. Fluorescence images were then captured using the Nikon T1 Eclipse microscope and NIS-Elements software (Nikon), at a magnification of ×40.

*Image J Analysis:* pS6 fluorescent outlines were generated using Image J on 10 independent images, for each individual cell line, across three independent biological replicates. Individual intensities were normalized to the area measured, and then a background intensity was subtracted from this value. Half of the average intensity of the DMSO treated group was used as a cutoff for p-S6 Low vs p-S6 High. The percent p-S6-low versus p-S6-high was calculated for each independent replicate and averaged across the biological replicates. Cleaved PARP positive nuclei for both p-S6-high and p-S6-low expressing cells were counted and calculated as a percentage of the total cell number for each treatment group, and averaged across the three independent experiments.

### Reverse Phase Protein Array

The RPPA assay was performed at the Functional Proteomics RPPA Core Facility at MD Anderson, as previously described^47^. Briefly, cells were seeded at a density of 0.3–0.5 × 10^6^ cells/well of a six-well plate in 3 ml of 5% RPMI and allowed to adhere overnight. Lysates were then collected, diluted in five 2-fold serial dilutions, and arrayed on nitrocellulose-coated slides (Grace Bio Lab) by Aushon 2470 Arrayer (Aushon BioSystems). Each slide was then probed with a primary antibody, followed by a biotin conjugated secondary antibody. Protein concentrations were then normalized for protein loading and corrected for by median centering across samples, and median centering across antibodies. Protein analysis was then performed at the MD Anderson Functional Proteomics RPPA Core Facility.

### Data Analysis

#### Elastic net regression analysis

Elastic net regression analysis (*α* = 0.8) was used to identify proteins, which expression (as measured by RPPA) is associated with the sensitivity and resistance of thyroid cancer cells to dasatinib. The regression analysis was performed with the help of the custom R script utilizing glmnet package^48^. Leave-one-out cross-validation was used to evaluate the modes and tuning parameter λ was selected to minimize the mean squared error on cross-validation.

#### Cluster analysis

Clustering of log transformed, centered and normalized and averaged expression data for phosphoproteins and tumor suppressors P16, RB, PTEN, and TP53 obtained with RPPA was performed with Cluster 3.0 and TreeView software. Spearman correlation distance metric and average linkage aggregation method were used. Cell lines were ordered by dasatinib IC_50_ (Fig. 1c)

### Statistical analysis

Experiments were performed with at least three separate replicates. Statistical analysis was performed using the GraphPad Prism software and the unpaired Student’s *t*-test was used to compare two means. Error bars represent the SEM, unless otherwise noted in respective figure legends.

## Electronic supplementary material

Supplemental Figure Legends

Supplemental Figure 1

Supplemental Figure 2

Supplemental Figure 3

Supplemental Figure 4

Supplemental Figure 5

Supplemental Table 1

Supplemental Table 2

Supplemental Table 3
