# Supplementary material for: Src-mediated regulation of the PI3K pathway in advanced papillary and anaplastic thyroid cancer
Source: Oncogenesis. 2018 Feb 28;7(2):23. doi: 10.1038/s41389-017-0015-5 (PMC5833015; doi:10.1038/s41389-017-0015-5)
Supplement: Supplementary file 1 — Supplemental Figure Legends [file 41389_2017_15_MOESM1_ESM.docx]

Supplemental Figure Legends

**Supplemental Figure 1: Leave-one-out cross-validation of the elastic net regression model of thyroid cancer dasatinib sensitivity.**  Tuning parameter λ is plotted on the x-axis. The optimal model (λ = -3.8, marked by dotted line) outperformed the null model (resulted in low mean-squared error).

**Supplemental Figure 2: Analysis of combined Src and MAPK pathway Inhibition. A.** Cleaved caspase 3/7 activity was measured after a 24 hour incubation with either DMSO, 100 nM trametinib, 100 nM dasatinib, or dasatinib + trametinib in the OCUT-2 and K1 cell line. Data as means +/- SEM B. Densitometry quantification of rpS6 phosphorylation at S235/236 and S240/244 in BCPAP, 8505C, T238, Cal62, C643, and THJ16T cell lines following a 24 hour treatment with 100nM trametinib, 100nM dasatinib, the combination, or an equivalent amount of DMSO. Quantification is based on at least 3 independent replicates for each cell line. Data as means +/- SEM (n=3; Student’s t-test; *, P < 0.05).

**Supplemental Figure 3. Increased tumor responsiveness correlates with a decrease in rpS6 phosphorylation.** Cal62 parental tumors derived from cells injected into the left and right flanks of Athymic Nude-Foxn1nu mice treated with either vehicle, 0.5mg/kg QD trametinib, 12.5mg/kg BD dasatinib, or the combination were harvested after 42 days of treatment, as described previously ^10^. Tumors were homogenized and whole cell lysates were analyzed by western blot analysis for the indicated antibodies. Densitometry was averaged across each treatment group. Data as means +/- SEM (n=4-8; Student’s t-test; *, P < 0.05).

**Supplemental Figure 4: rpS6 phosphorylation status in relation to the inhibition of Src and the MAPK pathway.** A. Densitometry quantification of rpS6 phosphorylation at S235/236 and S240/S244 in the 8505C and C643 cell lines expressing either expressing the doxycycline inducible P70S6K construct following a 24 hour treatment with 100nM trametinib, 100nM dasatinib, the combination, or an equivalent amount of DMSO. B. Colony area signal intensity was measured using Odyssey CLx imager (Li-Cor), and presented as percent fold change relative to the DMSO treated wells. Data as means +/- SEM (n=3). C. Densitometry quantification of rpS6 phosphorylation at S235/236 and S240/S244 in the 8505C and C643 parental cell lines treated with doxycycline prior to a 24 hour treatment with 100nM trametinib, 100nM dasatinib, the combination, or an equivalent amount of DMSO. D. Densitometry quantification of rpS6 phosphorylation at S235/S236 in the 8505C and C643 following a 24 hour treatment with 100nM trametinib, 100nM dasatinib, the combination, 1 µM everolimus or an equivalent amount of DMSO. E. Quantification of clonogenic growth following a 6 day treatment with 100nM trametinib, 100nM dasatinib, the combination, 1 µM everolimus or an equivalent amount of DMSO. The cells were then washed and released from treatment for 7 days. Quantification is based on at least 3 independent replicates for each cell line. Data as means +/- SEM (n=3; Student’s t-test; *, P < 0.05).

**Supplemental Figure 5: Combined inhibition of Src, MEK1/2, and AKT/P70S6K.** A. Densitometry quantification of rpS6 phosphorylation at S235/236 and S240/S244 in the 8505C and C643 cell lines following a 4 hour treatment with 100nM trametinib, 100nM dasatinib, Dasatinib + trametinib, 2.5 µM AT7867, AT7867 + trametinib, AT7867 + dasatinib, AT7867 + dasatinib + trametinib, or an equivalent amount of DMSO. Data as means +/- SEM (n=3). B. The cells were treated with either DMSO, 100nM trametinib, 100nM dasatinib, 2.5μM AT7867, trametinib + AT7867, dasatinib + AT7867, or trametinib + dasatinib + AT7867 for 3 days. Following 3 days of treatment, the cells were released for an additional 6 days. Colony area signal intensity was measured using Odyssey CLx imager (Li-Cor), and presented as percent fold change relative to the DMSO treated wells. Data as means +/- SEM (n=3).
