# Supplementary figures and images for: Src-mediated regulation of the PI3K pathway in advanced papillary and anaplastic thyroid cancer
Source: Oncogenesis. 2018 Feb 28;7(2):23. doi: 10.1038/s41389-017-0015-5 (PMC5833015; doi:10.1038/s41389-017-0015-5)

## Slide 1
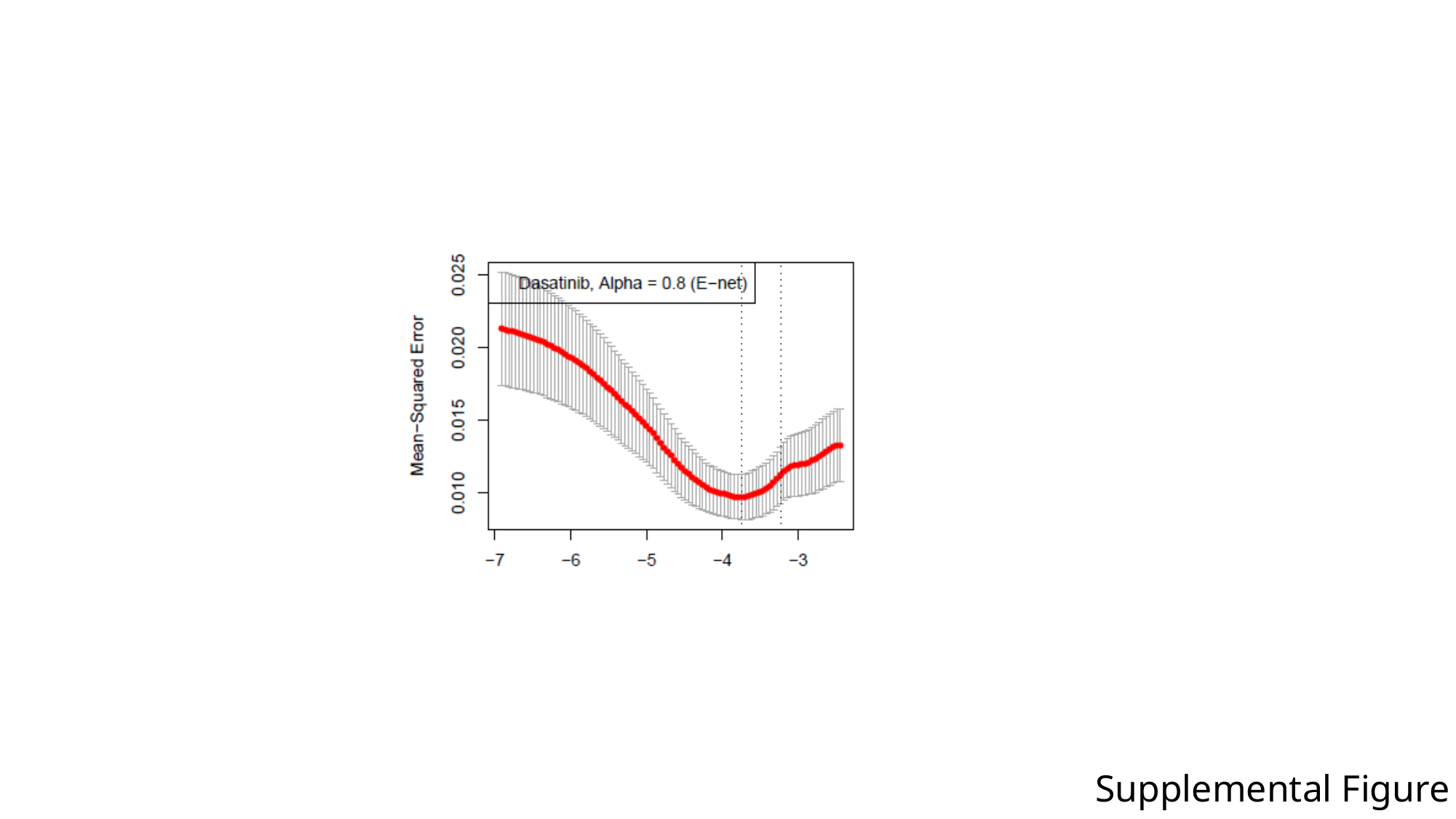

# Supplemental Figure 1

Supplement: Supplementary file 2 — Supplemental Figure 1 [file 41389_2017_15_MOESM2_ESM.pptx]
