# Supplementary material for: Src-mediated regulation of the PI3K pathway in advanced papillary and anaplastic thyroid cancer
Source: Oncogenesis. 2018 Feb 28;7(2):23. doi: 10.1038/s41389-017-0015-5 (PMC5833015; doi:10.1038/s41389-017-0015-5)
Supplement: Supplementary file 3 — Supplemental Figure 2 [file 41389_2017_15_MOESM3_ESM.pptx]

## Slide 1
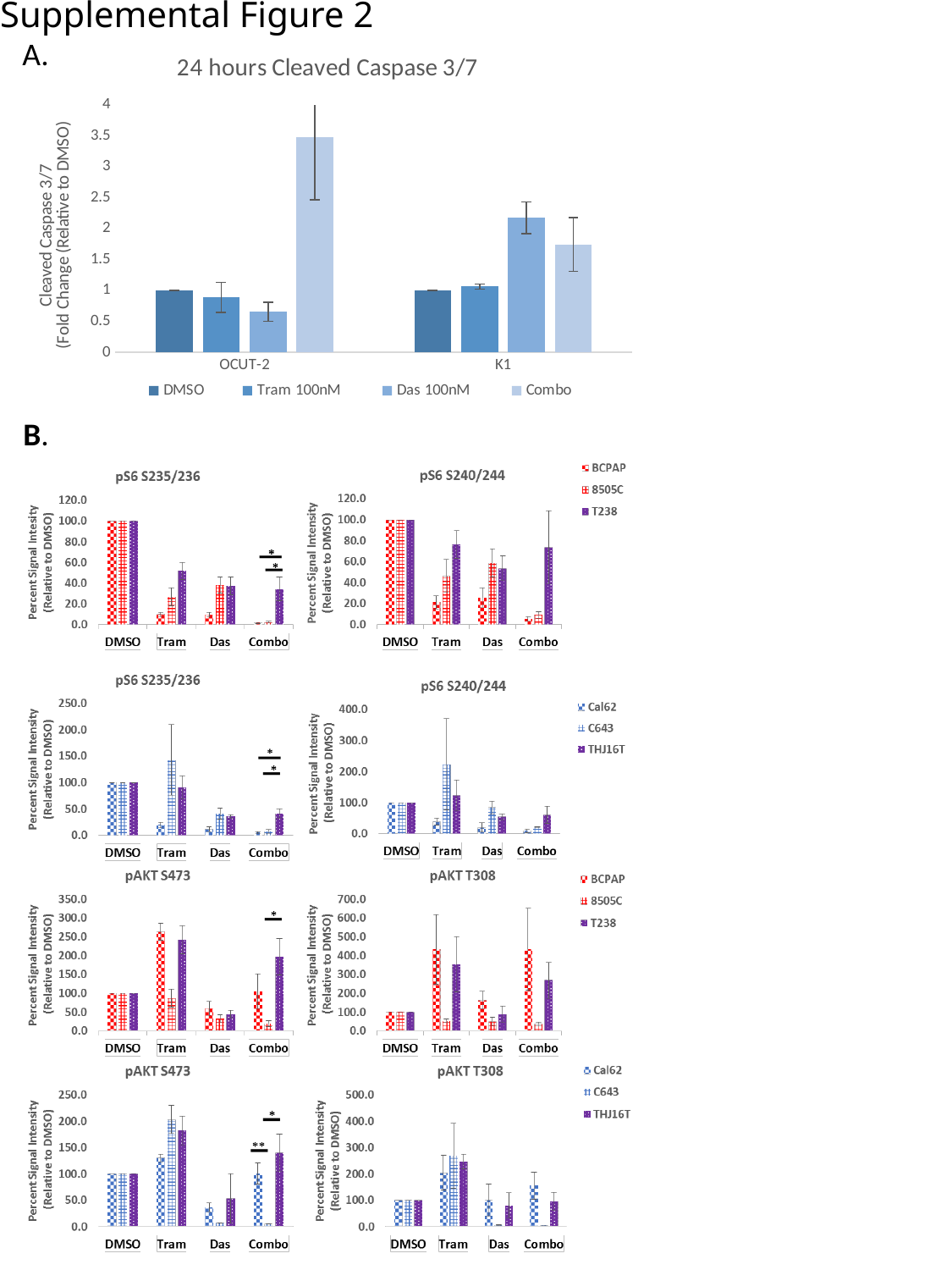

# Supplemental Figure 2
A.
### Chart: 24 hours Cleaved Caspase 3/7
| Category | DMSO | Tram 100nM | Das 100nM | Combo |
|---|---|---|---|---|
| OCUT-2 | 1.0 | 0.8881713979560127 | 0.65499687900616 | 3.479677625022887 |
| K1 | 1.0 | 1.065644349108916 | 2.177151580740464 | 1.746700515645692 |B.
