# Supplementary material for: Src-mediated regulation of the PI3K pathway in advanced papillary and anaplastic thyroid cancer
Source: Oncogenesis. 2018 Feb 28;7(2):23. doi: 10.1038/s41389-017-0015-5 (PMC5833015; doi:10.1038/s41389-017-0015-5)
Supplement: Supplementary file 4 — Supplemental Figure 3 [file 41389_2017_15_MOESM4_ESM.pptx]

## Slide 1
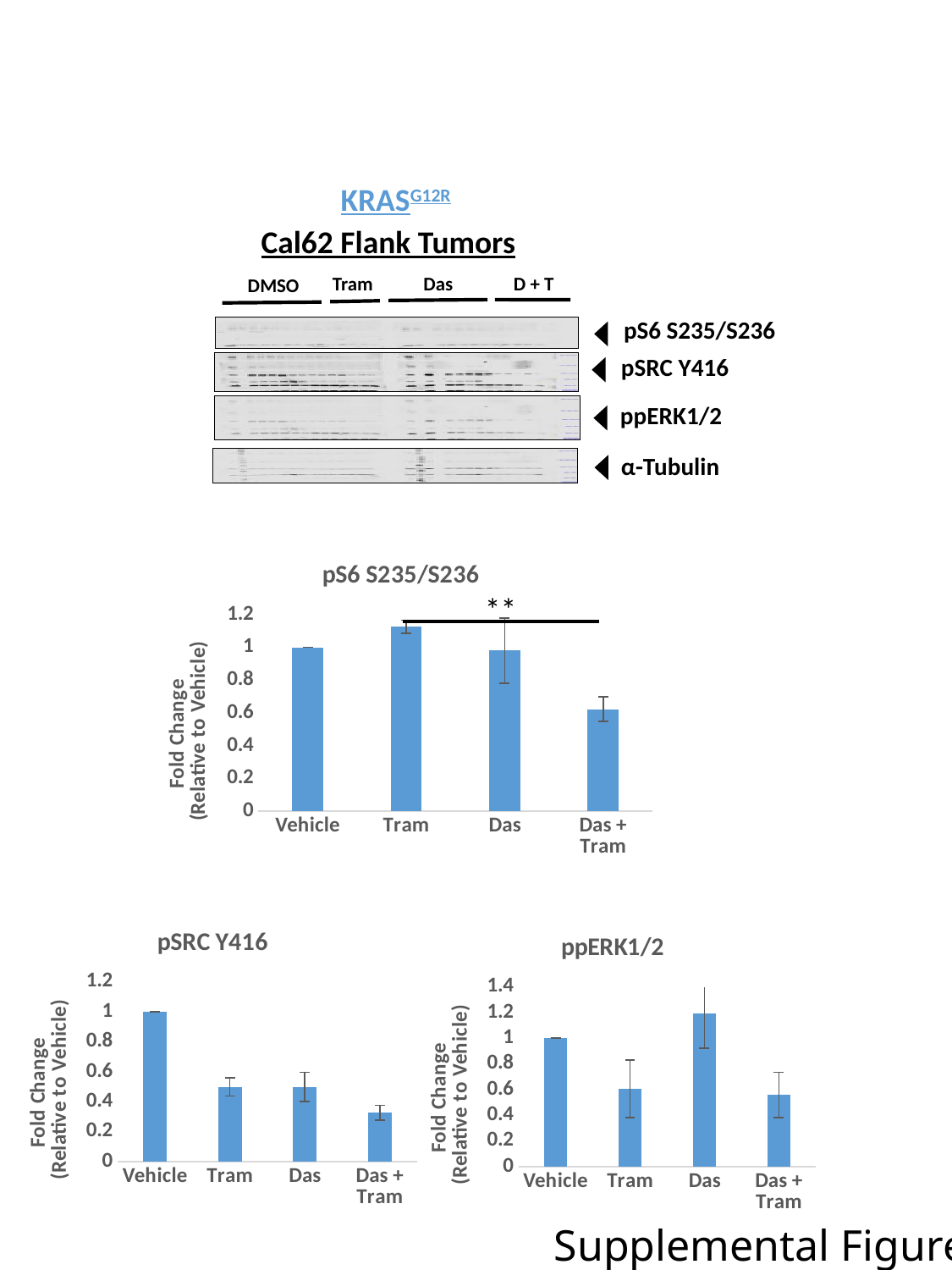

KRASG12R
Cal62 Flank Tumors
D + T
Das
Tram
DMSO
pS6 S235/S236
pSRC Y416
ppERK1/2
α-Tubulin
### Chart: pS6 S235/S236
| Category | |
|---|---|
| Vehicle | 1.0 |
| Tram | 1.1261826859054342 |
| Das | 0.9799719504932907 |
| Das + Tram | 0.6227054909626999 |**
### Chart: pSRC Y416
| Category | pERK |
|---|---|
| Vehicle | 1.0 |
| Tram | 0.4968693754215271 |
| Das | 0.496955297382041 |
| Das + Tram | 0.3254243105453978 |
### Chart: ppERK1/2
| Category | pERK |
|---|---|
| Vehicle | 1.0 |
| Tram | 0.6050998155554701 |
| Das | 1.1924153151122503 |
| Das + Tram | 0.5581320010113419 |# Supplemental Figure 3
