# Supplementary material for: Src-mediated regulation of the PI3K pathway in advanced papillary and anaplastic thyroid cancer
Source: Oncogenesis. 2018 Feb 28;7(2):23. doi: 10.1038/s41389-017-0015-5 (PMC5833015; doi:10.1038/s41389-017-0015-5)
Supplement: Supplementary file 5 — Supplemental Figure 4 [file 41389_2017_15_MOESM5_ESM.pptx]

## Slide 1
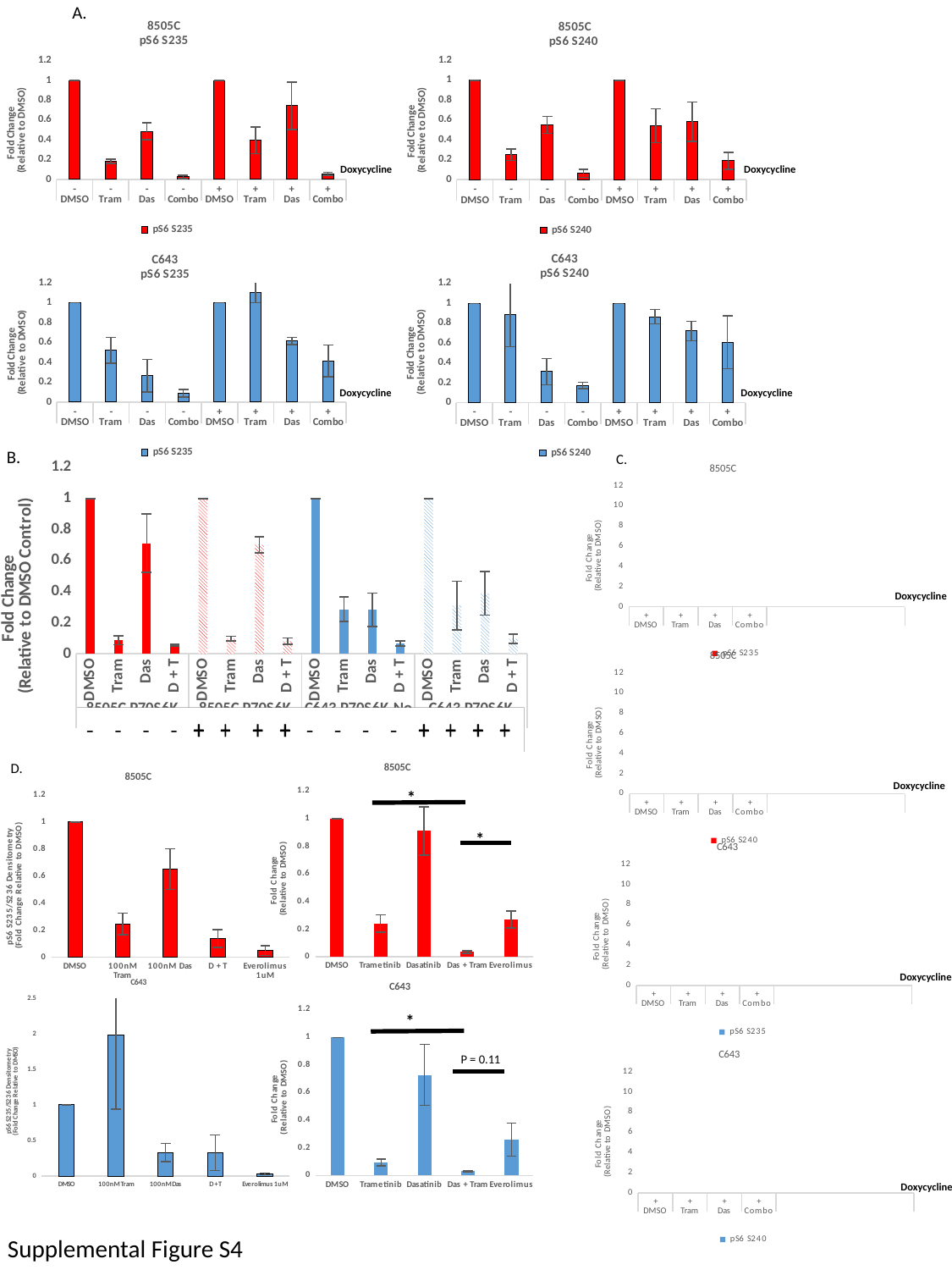

A.
### Chart: 8505C
pS6 S240
| Category | pS6 S240 |
|---|---|
| - | 1.0 |
| - | 0.24994816413979057 |
| - | 0.5511465105789165 |
| - | 0.06100159389055571 |
| + | 1.0 |
| + | 0.5421897036149047 |
| + | 0.5834260945124279 |
| + | 0.1893718546019595 |
### Chart: 8505C
pS6 S235
| Category | pS6 S235 |
|---|---|
| - | 1.0 |
| - | 0.18355463414392603 |
| - | 0.48573547394386835 |
| - | 0.026494278261974567 |
| + | 1.0 |
| + | 0.3945781202603896 |
| + | 0.7444863322765177 |
| + | 0.05586750973584232 |
### Chart: C643
pS6 S235
| Category | pS6 S235 |
|---|---|
| - | 1.0 |
| - | 0.5210653935232971 |
| - | 0.2653344897633317 |
| - | 0.08855787840971711 |
| + | 1.0 |
| + | 1.1064985595166013 |
| + | 0.6161396702114363 |
| + | 0.41423828251489603 |
### Chart: C643
pS6 S240
| Category | pS6 S240 |
|---|---|
| - | 1.0 |
| - | 0.8859438595494676 |
| - | 0.31241756414885025 |
| - | 0.17139854041366967 |
| + | 1.0 |
| + | 0.8654530831192112 |
| + | 0.7223835585657237 |
| + | 0.6079069065469959 |Doxycycline
Doxycycline
Doxycycline
Doxycycline
B.
C.
### Chart: 8505C
| Category | pS6 S235 |
|---|---|
| + | 1.0 |
| + | 0.372632058601861 |
| + | 0.3436097536596717 |
| + | 0.0730061820629996 |
### Chart: 8505C
| Category | pS6 S240 |
|---|---|
| + | 1.0 |
| + | 1.1665295420709272 |
| + | 0.9247539600104348 |
| + | 0.3783865351336301 |
### Chart: C643
| Category | pS6 S235 |
|---|---|
| + | 1.0 |
| + | 0.9540976523332516 |
| + | 0.20784596823070153 |
| + | 0.11383163520372608 |
### Chart: C643
| Category | pS6 S240 |
|---|---|
| + | 1.0 |
| + | 0.8002768868412944 |
| + | 0.3883555857150838 |
| + | 0.2550515985794973 |
### Chart
| Category | Average Fold |
|---|---|
| DMSO | 1.0 |
| Tram | 0.0871823697598561 |
| Das | 0.7134308970624528 |
| D + T | 0.055268069648648735 |
| DMSO | 1.0 |
| Tram | 0.09708256999096891 |
| Das | 0.7031632907581817 |
| D + T | 0.08087163978841441 |
| DMSO | 1.0 |
| Tram | 0.28747999112014677 |
| Das | 0.28345843577712904 |
| D + T | 0.06636250498657172 |
| DMSO | 1.0 |
| Tram | 0.31125702818025913 |
| Das | 0.38968856369334925 |
| D + T | 0.09672969036108937 |
Doxycycline
D.
### Chart:
| Category | 8505C |
|---|---|
| DMSO | 1.0 |
| Trametinib | 0.2407651953784566 |
| Dasatinib | 0.9133121279521252 |
| Das + Tram | 0.03616034378680978 |
| Everolimus | 0.2702167734717879 |
### Chart: 8505C
| Category | pS6 S235/S236 |
|---|---|
| DMSO | 1.0 |
| 100nM Tram | 0.24584633195151417 |
| 100nM Das | 0.6516868032355672 |
| D + T | 0.13796404572371995 |
| Everolimus 1uM | 0.05003496202389863 |
*
*
### Chart: C643
| Category | pS6 S235/S236 |
|---|---|
| DMSO | 1.0 |
| 100nM Tram | 1.9825619154144676 |
| 100nM Das | 0.32974704155615303 |
| D + T | 0.3293322848697359 |
| Everolimus 1uM | 0.031806110837759465 |
### Chart:
| Category | C643 |
|---|---|
| DMSO | 1.0 |
| Trametinib | 0.09498292631768047 |
| Dasatinib | 0.728700347757663 |
| Das + Tram | 0.029886984827172114 |
| Everolimus | 0.25872410580309524 |*
P = 0.11
Doxycycline
Doxycycline
Doxycycline
# Supplemental Figure S4
