# Supplementary material for: Src-mediated regulation of the PI3K pathway in advanced papillary and anaplastic thyroid cancer
Source: Oncogenesis. 2018 Feb 28;7(2):23. doi: 10.1038/s41389-017-0015-5 (PMC5833015; doi:10.1038/s41389-017-0015-5)
Supplement: Supplementary file 6 — Supplemental Figure 5 [file 41389_2017_15_MOESM6_ESM.pptx]

## Slide 1
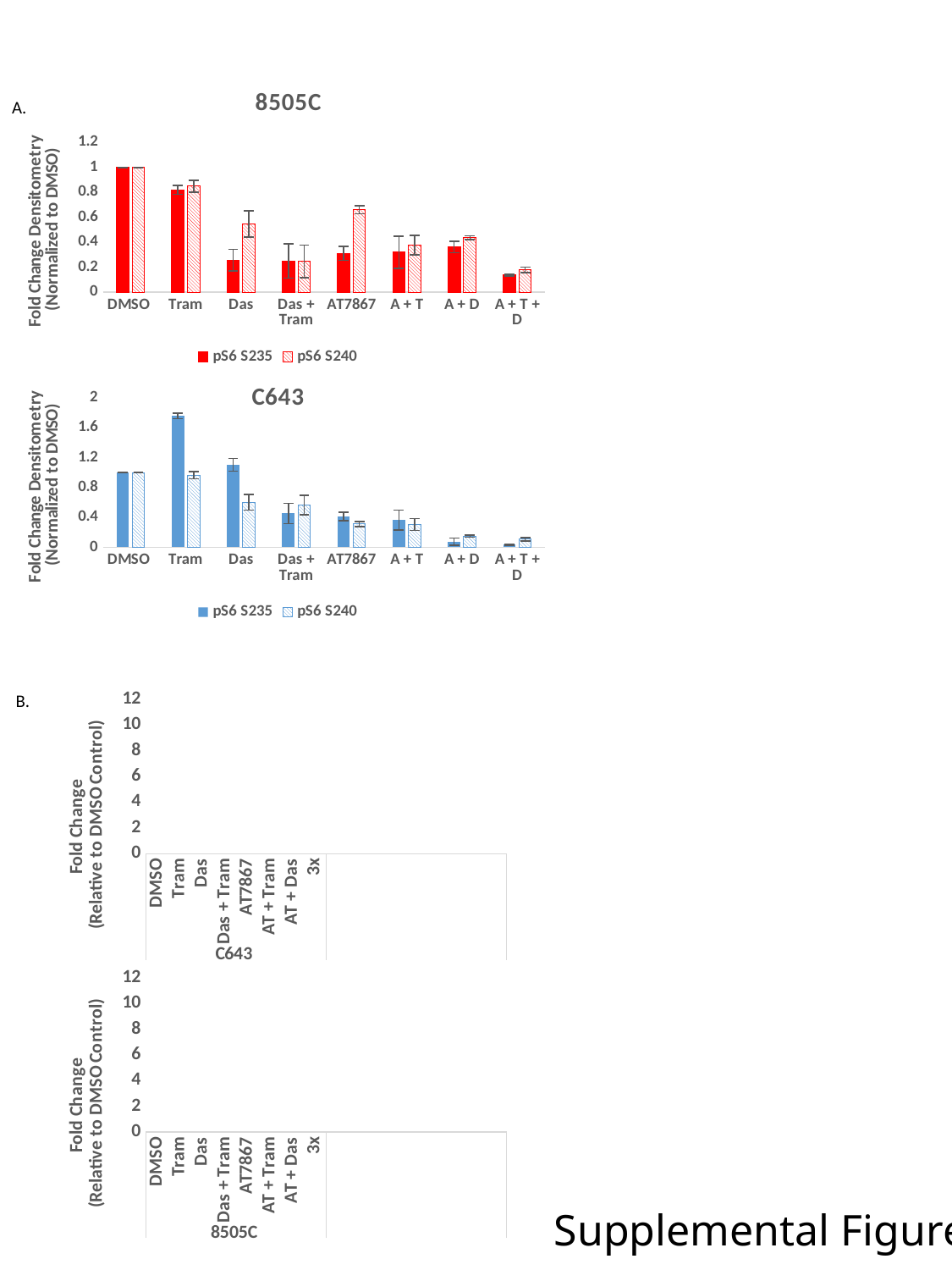

### Chart: 8505C
| Category | pS6 S235 | pS6 S240 |
|---|---|---|
| DMSO | 1.0 | 1.0 |
| Tram | 0.8208684527868273 | 0.8493244017419229 |
| Das | 0.25641212185654816 | 0.5480073200053956 |
| Das + Tram | 0.24916673719941393 | 0.24537895410483027 |
| AT7867 | 0.3093191305078047 | 0.6601932845920411 |
| A + T | 0.3190877424563528 | 0.3767615332505465 |
| A + D | 0.36278394105011286 | 0.4357706417759883 |
| A + T + D | 0.13602941916642702 | 0.17832135320589573 |
### Chart: C643
| Category | pS6 S235 | pS6 S240 |
|---|---|---|
| DMSO | 1.0 | 1.0 |
| Tram | 1.7572649136556544 | 0.9641264208196022 |
| Das | 1.1037915132485692 | 0.6006287737788795 |
| Das + Tram | 0.45220298133902725 | 0.5674787884978318 |
| AT7867 | 0.41185826731211006 | 0.31229574006893546 |
| A + T | 0.3647702871840803 | 0.30686614521841793 |
| A + D | 0.07587519950026711 | 0.15009802862627222 |
| A + T + D | 0.03530565266521885 | 0.10823261944962825 |A.
B.
### Chart
| Category | |
|---|---|
| DMSO | 1.0196783630537394 |
| Tram | 0.3432264398483962 |
| Das | 0.9127070862874695 |
| Das + Tram | 0.12641305195779343 |
| AT7867 | 0.40320676299642116 |
| AT + Tram | 0.1762969813231216 |
| AT + Das | 0.19470161636072547 |
| 3x | 0.07718136999657406 |
### Chart
| Category | |
|---|---|
| DMSO | 1.047353557225335 |
| Tram | 0.357263114476157 |
| Das | 1.148590131611659 |
| Das + Tram | 0.22735481090477433 |
| AT7867 | 0.5863609201664628 |
| AT + Tram | 0.16095618923710542 |
| AT + Das | 0.29586539232185377 |
| 3x | 0.06949768961253174 |# Supplemental Figure 5
