# Supplementary material for: Src-mediated regulation of the PI3K pathway in advanced papillary and anaplastic thyroid cancer
Source: Oncogenesis. 2018 Feb 28;7(2):23. doi: 10.1038/s41389-017-0015-5 (PMC5833015; doi:10.1038/s41389-017-0015-5)
Supplement: Supplementary file 7 — Supplemental Table 1 [file 41389_2017_15_MOESM7_ESM.pptx]

## Slide 1
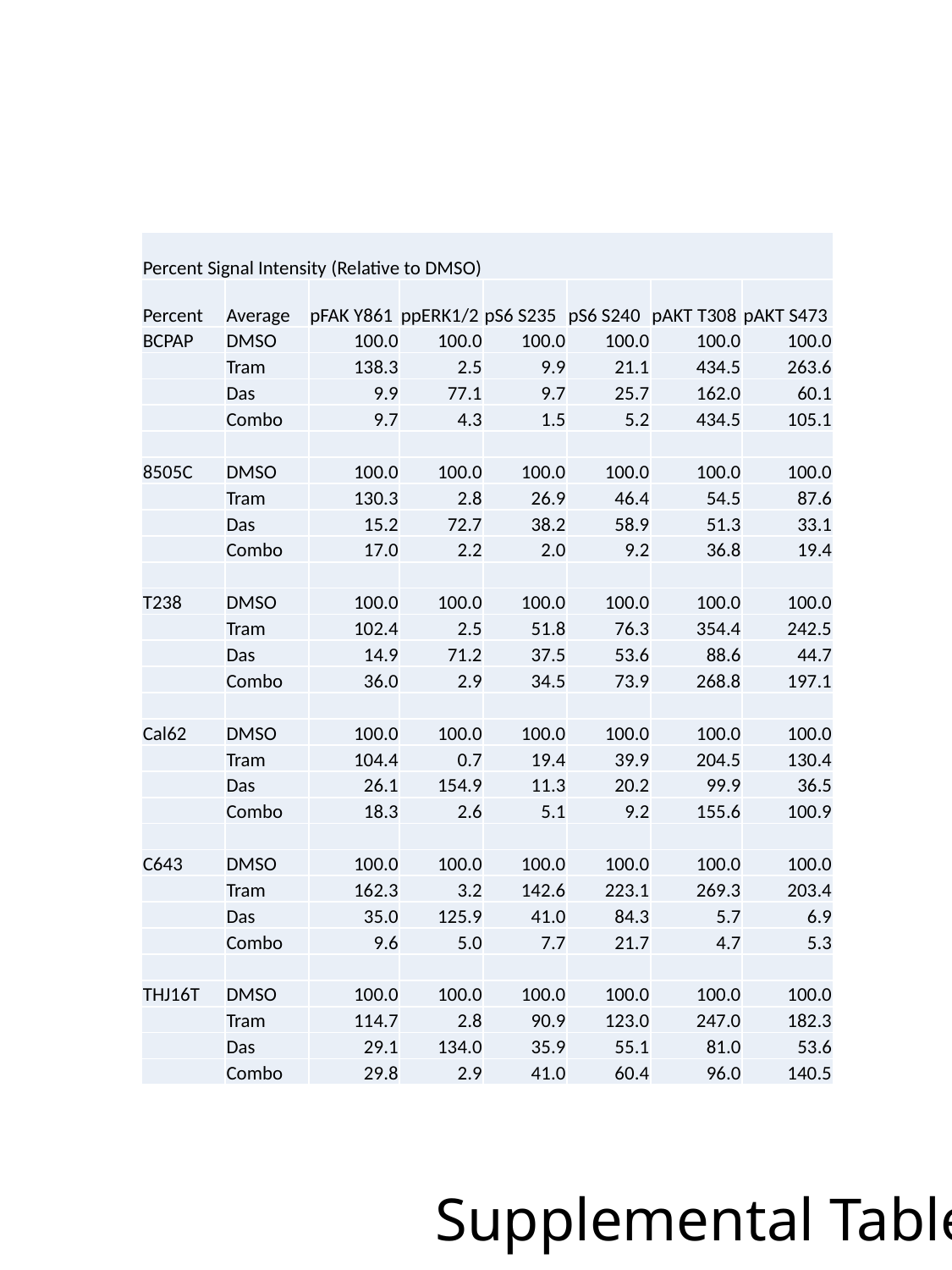

| Percent Signal Intensity (Relative to DMSO) | | | | | | | |
| --- | --- | --- | --- | --- | --- | --- | --- |
| Percent | Average | pFAK Y861 | ppERK1/2 | pS6 S235 | pS6 S240 | pAKT T308 | pAKT S473 |
| BCPAP | DMSO | 100.0 | 100.0 | 100.0 | 100.0 | 100.0 | 100.0 |
| | Tram | 138.3 | 2.5 | 9.9 | 21.1 | 434.5 | 263.6 |
| | Das | 9.9 | 77.1 | 9.7 | 25.7 | 162.0 | 60.1 |
| | Combo | 9.7 | 4.3 | 1.5 | 5.2 | 434.5 | 105.1 |
| | | | | | | | |
| 8505C | DMSO | 100.0 | 100.0 | 100.0 | 100.0 | 100.0 | 100.0 |
| | Tram | 130.3 | 2.8 | 26.9 | 46.4 | 54.5 | 87.6 |
| | Das | 15.2 | 72.7 | 38.2 | 58.9 | 51.3 | 33.1 |
| | Combo | 17.0 | 2.2 | 2.0 | 9.2 | 36.8 | 19.4 |
| | | | | | | | |
| T238 | DMSO | 100.0 | 100.0 | 100.0 | 100.0 | 100.0 | 100.0 |
| | Tram | 102.4 | 2.5 | 51.8 | 76.3 | 354.4 | 242.5 |
| | Das | 14.9 | 71.2 | 37.5 | 53.6 | 88.6 | 44.7 |
| | Combo | 36.0 | 2.9 | 34.5 | 73.9 | 268.8 | 197.1 |
| | | | | | | | |
| Cal62 | DMSO | 100.0 | 100.0 | 100.0 | 100.0 | 100.0 | 100.0 |
| | Tram | 104.4 | 0.7 | 19.4 | 39.9 | 204.5 | 130.4 |
| | Das | 26.1 | 154.9 | 11.3 | 20.2 | 99.9 | 36.5 |
| | Combo | 18.3 | 2.6 | 5.1 | 9.2 | 155.6 | 100.9 |
| | | | | | | | |
| C643 | DMSO | 100.0 | 100.0 | 100.0 | 100.0 | 100.0 | 100.0 |
| | Tram | 162.3 | 3.2 | 142.6 | 223.1 | 269.3 | 203.4 |
| | Das | 35.0 | 125.9 | 41.0 | 84.3 | 5.7 | 6.9 |
| | Combo | 9.6 | 5.0 | 7.7 | 21.7 | 4.7 | 5.3 |
| | | | | | | | |
| THJ16T | DMSO | 100.0 | 100.0 | 100.0 | 100.0 | 100.0 | 100.0 |
| | Tram | 114.7 | 2.8 | 90.9 | 123.0 | 247.0 | 182.3 |
| | Das | 29.1 | 134.0 | 35.9 | 55.1 | 81.0 | 53.6 |
| | Combo | 29.8 | 2.9 | 41.0 | 60.4 | 96.0 | 140.5 |
Supplemental Table 1
