# Supplementary material for: Src-mediated regulation of the PI3K pathway in advanced papillary and anaplastic thyroid cancer
Source: Oncogenesis. 2018 Feb 28;7(2):23. doi: 10.1038/s41389-017-0015-5 (PMC5833015; doi:10.1038/s41389-017-0015-5)
Supplement: Supplementary file 8 — Supplemental Table 2 [file 41389_2017_15_MOESM8_ESM.pptx]

## Slide 1
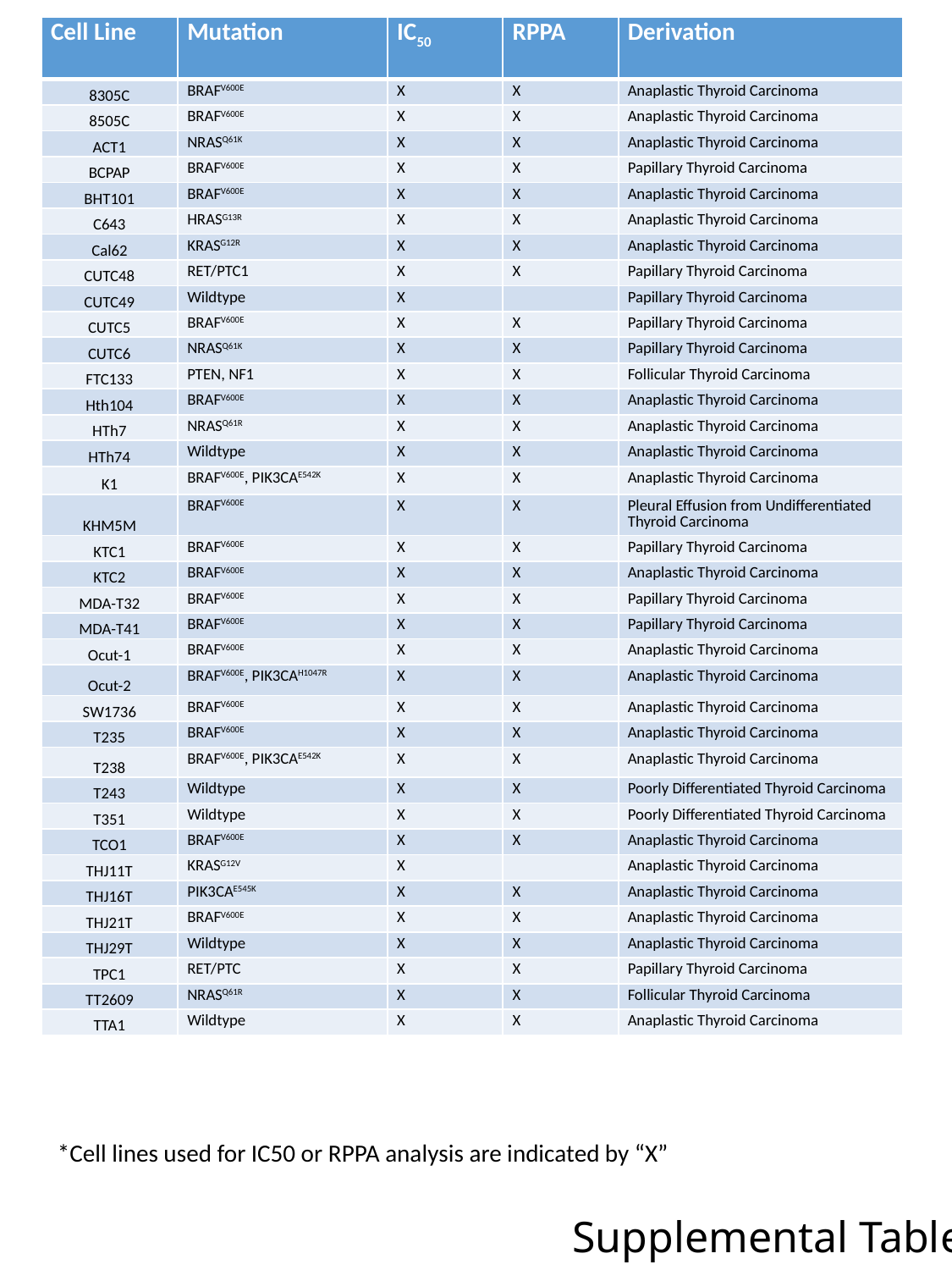

| Cell Line | Mutation | IC50 | RPPA | Derivation |
| --- | --- | --- | --- | --- |
| 8305C | BRAFV600E | X | X | Anaplastic Thyroid Carcinoma |
| 8505C | BRAFV600E | X | X | Anaplastic Thyroid Carcinoma |
| ACT1 | NRASQ61K | X | X | Anaplastic Thyroid Carcinoma |
| BCPAP | BRAFV600E | X | X | Papillary Thyroid Carcinoma |
| BHT101 | BRAFV600E | X | X | Anaplastic Thyroid Carcinoma |
| C643 | HRASG13R | X | X | Anaplastic Thyroid Carcinoma |
| Cal62 | KRASG12R | X | X | Anaplastic Thyroid Carcinoma |
| CUTC48 | RET/PTC1 | X | X | Papillary Thyroid Carcinoma |
| CUTC49 | Wildtype | X | | Papillary Thyroid Carcinoma |
| CUTC5 | BRAFV600E | X | X | Papillary Thyroid Carcinoma |
| CUTC6 | NRASQ61K | X | X | Papillary Thyroid Carcinoma |
| FTC133 | PTEN, NF1 | X | X | Follicular Thyroid Carcinoma |
| Hth104 | BRAFV600E | X | X | Anaplastic Thyroid Carcinoma |
| HTh7 | NRASQ61R | X | X | Anaplastic Thyroid Carcinoma |
| HTh74 | Wildtype | X | X | Anaplastic Thyroid Carcinoma |
| K1 | BRAFV600E, PIK3CAE542K | X | X | Anaplastic Thyroid Carcinoma |
| KHM5M | BRAFV600E | X | X | Pleural Effusion from Undifferentiated Thyroid Carcinoma |
| KTC1 | BRAFV600E | X | X | Papillary Thyroid Carcinoma |
| KTC2 | BRAFV600E | X | X | Anaplastic Thyroid Carcinoma |
| MDA-T32 | BRAFV600E | X | X | Papillary Thyroid Carcinoma |
| MDA-T41 | BRAFV600E | X | X | Papillary Thyroid Carcinoma |
| Ocut-1 | BRAFV600E | X | X | Anaplastic Thyroid Carcinoma |
| Ocut-2 | BRAFV600E, PIK3CAH1047R | X | X | Anaplastic Thyroid Carcinoma |
| SW1736 | BRAFV600E | X | X | Anaplastic Thyroid Carcinoma |
| T235 | BRAFV600E | X | X | Anaplastic Thyroid Carcinoma |
| T238 | BRAFV600E, PIK3CAE542K | X | X | Anaplastic Thyroid Carcinoma |
| T243 | Wildtype | X | X | Poorly Differentiated Thyroid Carcinoma |
| T351 | Wildtype | X | X | Poorly Differentiated Thyroid Carcinoma |
| TCO1 | BRAFV600E | X | X | Anaplastic Thyroid Carcinoma |
| THJ11T | KRASG12V | X | | Anaplastic Thyroid Carcinoma |
| THJ16T | PIK3CAE545K | X | X | Anaplastic Thyroid Carcinoma |
| THJ21T | BRAFV600E | X | X | Anaplastic Thyroid Carcinoma |
| THJ29T | Wildtype | X | X | Anaplastic Thyroid Carcinoma |
| TPC1 | RET/PTC | X | X | Papillary Thyroid Carcinoma |
| TT2609 | NRASQ61R | X | X | Follicular Thyroid Carcinoma |
| TTA1 | Wildtype | X | X | Anaplastic Thyroid Carcinoma |
# Supplemental Table 2
*Cell lines used for IC50 or RPPA analysis are indicated by “X”
