# Supplementary material for: Src-mediated regulation of the PI3K pathway in advanced papillary and anaplastic thyroid cancer
Source: Oncogenesis. 2018 Feb 28;7(2):23. doi: 10.1038/s41389-017-0015-5 (PMC5833015; doi:10.1038/s41389-017-0015-5)
Supplement: Supplementary file 9 — Supplemental Table 3 [file 41389_2017_15_MOESM9_ESM.pptx]

## Slide 1
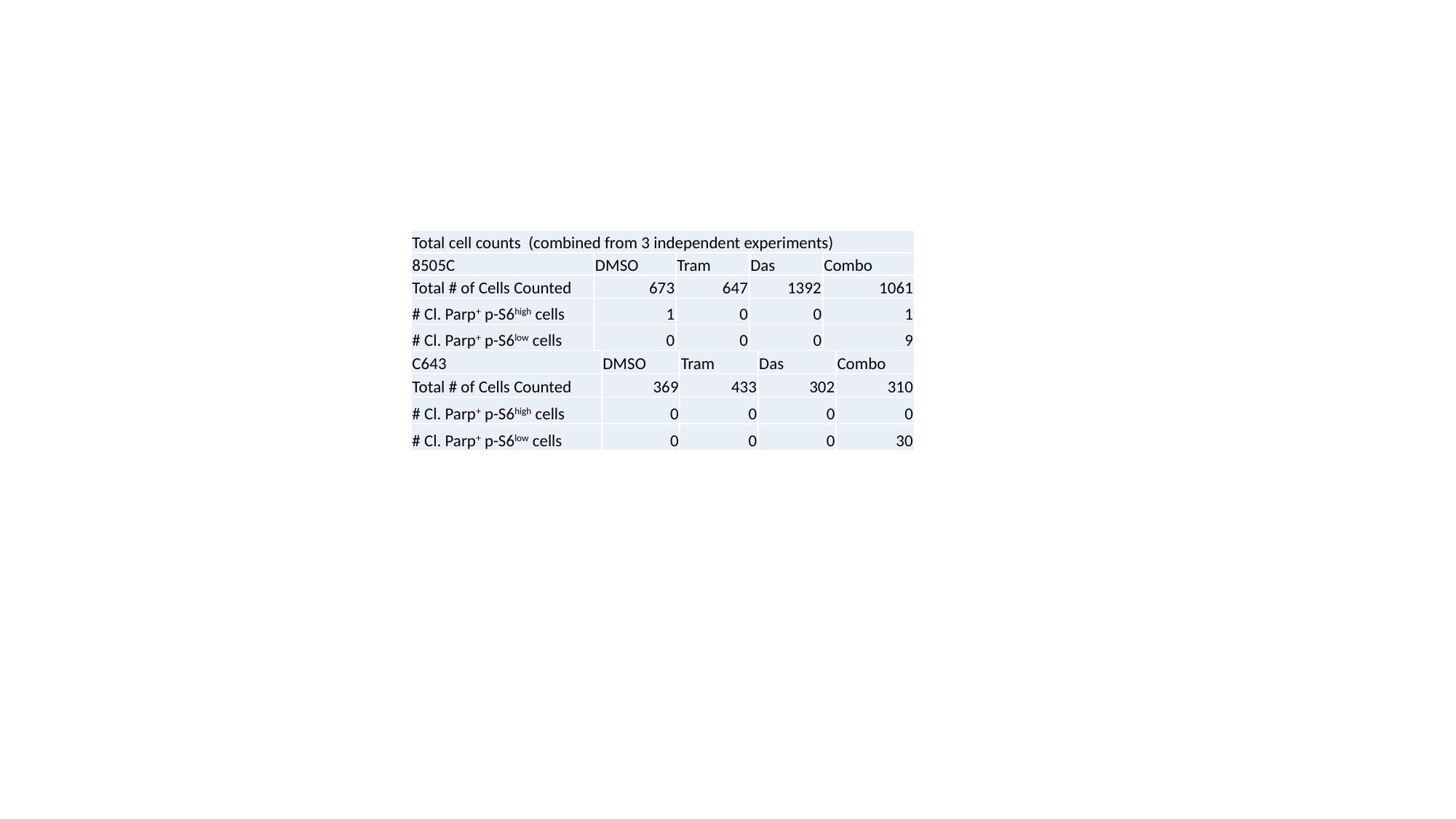

| Total cell counts (combined from 3 independent experiments) | | | | |
| --- | --- | --- | --- | --- |
| 8505C | DMSO | Tram | Das | Combo |
| Total # of Cells Counted | 673 | 647 | 1392 | 1061 |
| # Cl. Parp+ p-S6high cells | 1 | 0 | 0 | 1 |
| # Cl. Parp+ p-S6low cells | 0 | 0 | 0 | 9 |
| C643 | DMSO | Tram | Das | Combo |
| --- | --- | --- | --- | --- |
| Total # of Cells Counted | 369 | 433 | 302 | 310 |
| # Cl. Parp+ p-S6high cells | 0 | 0 | 0 | 0 |
| # Cl. Parp+ p-S6low cells | 0 | 0 | 0 | 30 |
